# Asymmetry of Musculature and Hand Grip Strength in Bodybuilders and Martial Artists

**DOI:** 10.3390/ijerph17134695

**Published:** 2020-06-30

**Authors:** Anna Burdukiewicz, Jadwiga Pietraszewska, Justyna Andrzejewska, Krystyna Chromik, Aleksandra Stachoń

**Affiliations:** Department of Physical Anthropology, University School of Physical Education, 51-612 Wrocław, Poland; jadwiga.pietraszewska@awf.wroc.pl (J.P.); justyna.andrzejewska@awf.wroc.pl (J.A.); krystyna.chromik@awf.wroc.pl (K.C.); aleksandra.stachon@awf.wroc.pl (A.S.)

**Keywords:** bilateral asymmetry, muscle mass, hand grip strength, bodybuilding, jiu-jitsu, judo

## Abstract

The functional preference for the upper limb influences the occurrence of bilateral differences in other segments of the human body. The aim of the study is to assess the influence of the applied fighting technique and targeted physical effort on the occurrence of asymmetry in body musculature and isometric strength in bodybuilders and competitors of selected martial arts. Academic athletes practicing judo (J), jiu-jitsu (JJ), and bodybuilding (BB) were examined. The control group (C) consisted of students who do not practice any sports. The assessment of the body structure was conducted through segmental bioelectrical impedance analysis. Moreover, the study took into account the measurements of left- and right-hand grip strength. In judo, the uneven physical exertion of the right and left sides of the body further increases both directional and absolute asymmetry. Bilateral asymmetry of musculature in jiu-jitsu competitors and bodybuilders occurs to a lesser extent. The control group was characterized by cross-asymmetry. So as to avoid the risk of injury of sportsmen, it is important to consistently supervise and correct their body structure, which also includes the symmetrical participation of the active muscle mass in particular segments. The symmetrisation process should be individualized since each particular sportsman has their own side-to-side body morphology.

## 1. Introduction

Humans are the only species among the primates that are characterized by directional asymmetry, which is caused by lateralization. It is manifested in their preference for the right upper limb [1]; this fact, in turn, means that bilateral discrepancies occur consistently across the population. In contrast, fluctuating asymmetry takes form of non-directional deviations from the expected symmetry. In addition, there is anti-symmetry, which is a non-systematic bilateral difference in the population. Directional asymmetry and antisymmetry are considered to be conditioned genetically to a certain extent, whereas fluctuating asymmetry is a manifestation of developmental instability [2,3].

It is estimated that the frequency of left-handedness equals 10–13%, although there is some degree of variance caused by socio-cultural and geographical factors [4]. Left-handedness appears to be quite advantageous in the context of one-on-one combat: A certain dose of unpredictable behavior may directly affect the outcome of a fight. The literature on the subject confirms this observation: The research has shown that left-handed sportsmen tend to be overrepresented on the professional level in the contact sports whereas no such dependence has been found in the non-contact sports [5].

In order to counteract the temporal and spatial limitations present in contact sports, the competitors develop their cognitive skills, perception, and motor abilities relevant to the actions they perform [6]. The prevalence of right-handed competitors may be causing the development of a skillset that allows sportsmen to better equipped against the right-handed opponents, rather than the left-handed [7]. This allows the competitors to be more effective at predicting the actions that the right-handed competitors intend to take whereas the actions of the left-handed competitors are more difficult to predict [8]. The research has also shown that the left-handed competitors also tend to perform better when under time pressure [9].

Vertebrates are characterized by behavioral lateralization, which manifests in directional asymmetry; the extent to which the asymmetry occurs is dictated by the differences in the mechanical strain that lead to deviations from perfect symmetry [10,11]. In humans, functional preference for the right upper limb means that the domination of the latter over the left upper limb in terms of the morphological features is very pronounced. Sports practice causes asymmetrical development of bones and musculature of the dominant limb [12]. Heavy labor can also impact the body in a similar way [13]. In turn, the asymmetry in palm size correlates with handedness [14]. Cross-asymmetry has also been observed within the upper and lower limbs, which may indicate that the asymmetrical use of the upper limbs may also impact the asymmetry of the lower limbs in the opposite direction [11]. The research has also shown a significant degree of morphological discrepancy within the pelvis, which indicates the domination of the left side of the lower body; this is in line with the models that describe the influence of right-handedness on the lower body [15]. The directional asymmetry within the lower limbs is less marked, especially in terms of their length; it is, however, visibly marked in terms of the width of diaphysis and epiphysis of the bones [9,16].

The degree of asymmetry is caused by both by the mechanical and genetic factors. Any strain caused by the performed jobs or systematic participating in workout influence the discrepancies in the distribution of morphological features on both sides of the body [17,18]. In the case of the younger competitors in whom epiphyseal closure has not occurred yet, excessive physical strain may cause further discrepancies as well [19]. In sports, the morphological discrepancies of the body sides also depend on the particular discipline that the sportsmen practice.

Moreover, in sports, participating in the disciplines that mainly involve a particular side of the body entails asymmetrical changes in certain tissues. These changes are visible especially in soft tissue [20]. The analysis of bilateral differences in javelin throwers has indicated that the asymmetry occurs in the upper limb circumference and is particularly visible in skinfold thickness [21]. The research has also confirmed the significant influence of sports practice on the asymmetry in the distribution of the upper limb muscle mass in young tennis players. In these sportsmen, the muscle mass was far greater in their dominant upper limb if compared to the non-dominant upper limb. Moreover, no significant correlation was found between the age of the sportsmen, the length of their sports practice experience, and the asymmetry coefficients [22].

The research has also shown that the level of asymmetry rises with the sports proficiency of the competitors. In professional field hockey players, both male and female, the research found a significant increase in the muscle mass and in bone mineral density on the left side of the body [23]. In football players, long-term routine physical strain influences the morphological structure of their lower limbs and, as a result, the expansion of the bone mass, the bone cross-sectional area and the thickness of the cortical layer of the bones [24]. The relationship has also been found between the accuracy of marksmen and the increase in their lean mass and the reduction in their fat mass. Moreover, the more efficient marksmen were also characterized by the lesser degree of asymmetry in the lean mass and the strength of their lower limbs [25]. Examination of sportsmen through the DXA method has shown the influence of asymmetry in the lean body mass of the lower limb on the asymmetry of strength and power when jumping [26]. In competitors participating in 100 m runs, a trend to perform better was found in the athletes with more symmetrical structure of their knee joints and ankle joints [27].

The research cited hereinabove shows the multitude of body asymmetries found in the sportsmen from various disciplines; these asymmetries are a result of one-sided strain during sports practice. Though it has not yet been sufficiently described in academic studies, martial arts are potentially an example of a discipline that involves one side of the body over the other. Moreover, this phenomenon should not be present in bodybuilding for it is a discipline that highly emphasizes the balanced modelling of one’s physique. The aim of the study is to assess the influence of the applied fighting techniques and targeted physical effort on the occurrence of asymmetry in body musculature and isometric strength in bodybuilders and competitors of selected martial arts.

## 2. Materials and Methods

The study examined 120 men aged 21.6 ± 2.6 who do sports in academic sports clubs at the major universities in Wrocław. The sportsmen belonged to the following disciplines: Judo (J), Brazilian jiu-jitsu (JJ), and the amateur group of natural bodybuilders (BB). The control group (C) consisted of students (*n* = 30) aged 20.8 ± 2.5 who do not practice any sport. All groups consisted of 30 participants each. Variance analysis was used to confirm that there were no significant age differences across the groups. However, statistically significant differences were present in the group members in terms of the length of their sports practice experience (judo 9.9 ± 3.2; ju-jitsu 4.8 ± 1.8; bodybuilding 3.7 ± 2.4).

All subjects gave their informed consent for inclusion before they participated in the study. The study was conducted in accordance with the Declaration of Helsinki, and the protocol was approved by the Ethics Committee of the University School of Physical Education in Wrocław, Poland (23.10.12). A survey was used to collect information regarding the participants’ date of birth, length of their sports practice experience, and preferred martial art techniques (in the case of judo and jiu-jitsu competitors) as well as any physical traumas they might have experienced. None of the athletes have declared an injury. Athletes previously reported that they did not use any exogenous anabolic androgenic steroids, drugs, medication, or dietary supplements. In all groups the frequency of left-handedness was about 12%.

All measurements were done during the morning. The body measurements were taken with the use of an anthropometer accurate to up to 0.1 cm (GPM Siber Hegner Machinery Ltd., Zurich Switzerland) by professional anthropologists who specialize in taking such measurements. The body mass was measured with the use of an electronic scale accurate up to 0.1 kg (Fawag, Lublin, Poland). The somatic features were used to assess the weight-height proportions. In order to do that, BMI was calculated (body mass [kg]/body height [m]^2^).

The research on structural asymmetry of limbs in sportsmen may involve expensive and time-consuming methods such as ultrasonography, MRI, and DXA [28]. However, these cannot be applied in field conditions. Therefore, in order to assess body structure, this research used electrical bioimpedance [29].

The device with in-built BodyScan module was used: BIA-101 Anniversary Sport Edition made by Akern (tetrapolar and octopolar version, electrode position: Hand–foot, BodyGram 1.31 software, BodyScan 5.0; Florence, Italy). Body structure measurements were taken in accordance with manufacturer’s recommendations (the subjects were in fasting condition during the measurements, they were lying on their backs in a horizontal position, their limbs were resting at a 40 degree angle from the body, and the time between the measurement and their last physical effort was 12 h or more). The analysis took the following elements of body structure into account: Fat mass, muscle mass, the size of muscle mass of the body segments (trunk, upper limb, lower limb) on both sides of the body, and the of muscle mass of left and right side of the body. The components of the body structure are expressed in both absolute values [kilograms] and in percentages.

Moreover, the study took into account the measurements of left- and right-hand grip strength, which is considered to be a strong predictor for success in martial arts [30]. Grip strength was measured with the use of the hand grip dynamometer (T.K.K.5001, Takei Scientific Inst. Co., Ltd., Niigata, Japan). The aim of this test was to measure the maximum isometric strength of the muscles in palm and forearm. During the measurement, the upper limb was straightened and lowered [31].

Statistical Methods

The calculations were carried out with the use of Statistica 13 package (Dell Inc., Tulsa, OK, United States) Shapiro–Wilk test was used to examine the distributions in the analyzed characteristics. Variance analysis and Tukey’s HSD test were used to assess the intergroup differences in body structure and in the analyzed anthropometric characteristics. The paired t-test was utilized to determine if right and left sides were significantly different for each dimension. Alpha level was set at *p* < 0.05.

Standardized directional asymmetry (DA) score is the quantitative measure of directional asymmetry in the size of musculature of particular body segments and grip strengths [10]. It is calculated as:

%DA = (R − L)/(1/2(R + L)) × 100(1)

where L = left measurement and R = right measurement. The scores above zero mean that the right-side measurements are higher whereas the scores below zero mean that the left-side measurements were higher. Standardized absolute asymmetry (AA) score is a measure that does not account for the directionality of the asymmetry and is calculated as follows:

%AA = (|R − L|)/(1/2(R + L)) × 100(2)

Since the DA and AA values deviated from the normal distribution, the non-parametric Kruskal–Wallis test was used.

Cluster analysis made it possible to re-arrange the structure of the tested variables. The distances in cluster analysis were calculated with the use or 1-r Pearson’s formula. Grouping was done with the use of Ward’s method [32]. This approach provides a structure that contains minimal variance within the clusters and maximum variation between the clusters. The dendrogram groups the variables that differ the least from one another and are connected on a given level of similarity. The division of the dendrogram was done by analyzing the consequences of dividing the taxonomic pyramid on different levels. The best choice is to divide the dendrogram at the height that precedes the significant decrease of probability between the objects or clusters.

## 3. Results

Intergroup difference of body height is statistically insignificant (Table 1). Bodybuilders are characterized by the greatest body height whereas the lowest height was found in jiu-jitsu competitors. However, the groups of subjects significantly differ from one another with regard to body mass and weight-height proportions. The non-training participants have significantly lower body mass than the judo competitors and the bodybuilders. The greatest BMI values were found in the judo competitors whereas the lowest were in the control group. Fat mass was significantly higher in the control group than in the judo group and the bodybuilder group. The percentage of muscle mass was significantly higher in the martial arts athletes and in the control group than in bodybuilder.

With regard to the muscle mass of the right and left side of the body and of the upper limb and the lower limb, the differences are statistically significant only in the comparison of the judokas and the control group. Bilateral differentiation of the musculature in the jiu-jitsu competitors and bodybuilders is statistically insignificant. The differences in the size of musculature of the right and left side of the trunk are also low.

In percentage terms, the muscle mass on the right and left side of the body is similar for all groups. The jiu-jitsukas and bodybuilders are characterized by a somewhat higher muscle mass on the right side of the body whereas in the judo athletes and non-sportsmen, this feature shows higher values on the left side of the body. The percentage of the muscles on the right and left upper and lower limbs is significantly higher in the judo competitors in comparison to the control group. The non-sportsmen have significantly higher total percentage volume of muscles on the right and left side of the trunk in comparison to the judokas. In all groups, grip strength of the right hand was higher than that of the left hand. The paired sample t-test showed no significant statistical difference in bilateral musculature development. The significantly higher results were found in the right-hand grip strength tests in the bodybuilders (difference = 4.7 sd = 4.46) and in the control group (difference = 2.8 sd = 3.64).

In the group of judo competitors, directional asymmetry is most marked in the musculature of the upper limbs and trunk (Table 2). Except for the upper limb, in this group, the size of muscle mass on the left side of the body is far higher than that on the right side. All the directional asymmetry markers have positive values in jiu-jitsu competitors and bodybuilders. In control group, the size of musculature was significantly higher for the right half of the body and the right upper limb. The muscle mass of the lower limb and trunk is much higher on the left side. In all groups, the DA score for grip strength indicates the prevalence of the right hand.

The absolute asymmetry markers that ignore the directionality are the highest in the judo group and lowest in the bodybuilders. Judo competitors also show the greatest differences in terms of the size of muscle mass of the right and left sides of the body. Only in jiu-jiutsukas, absolute asymmetry of musculature of the lower limb is higher if compared to the upper limb. Conversely, judo competitors are characterized by higher asymmetry in the size of muscle mass of the upper limb and the lower limb. Similarly, judokas rank highest in AA for the size of musculature of left and right side of the trunk. The absolute asymmetry index for grip strength is lowest in the jiu-jitsu athletes. AA for grip strength is highest in the bodybuilding group.

**Figure 1:**
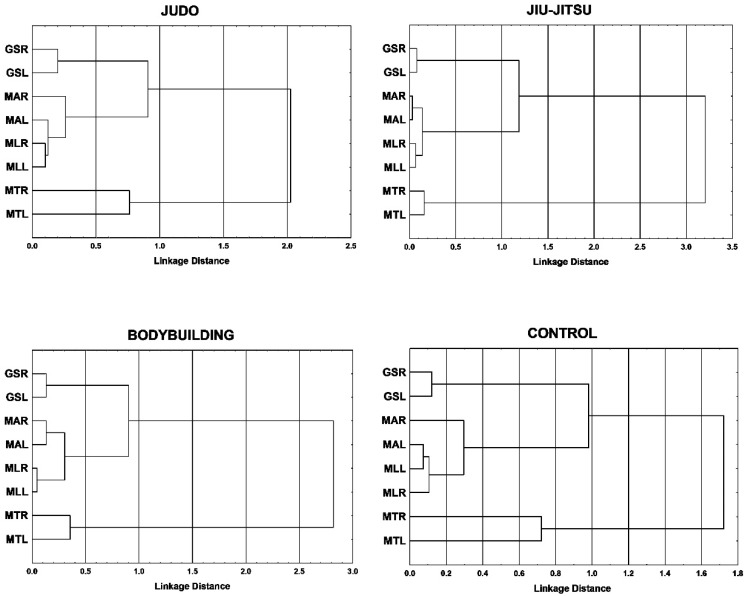
Cluster analysis of musculature of body segments and grip strength in martial arts athletes, bodybuilders, and control group. MTR—muscle trunk right; MTL—muscle trunk left; MLR—muscle leg right; MLL—muscle leg left; MAR—muscle arm right; MAL—muscle arm left; GSR—grip strength right; GSL—grip strength left.

Dendrogams illustrate hierarchical structure of the analyzed variables based on the decreasing similarity of those traits (Figure 1). In all groups, the measurements of grip strength constitute a separate cluster for which the agglomeration coefficient is low and does not exceed 0.5. Another separate cluster is formed by the muscle mass of the right and left side of the trunk but in its case, the agglomeration coefficient varies far more, i.e., 0.17 in the jiu-jitsukas and 0.76 in the judokas. In the control group, it equals 0.72 and in the bodybuilders: 0.33. The other characteristics constitute a separate, four-element cluster that comprises the variables of the musculature of the right and left upper and lower limb. In judo competitors, a strong relationship is visible between the musculature of the right and left lower limb to which the musculature of the left upper limb is adjoined. Further in the graph, these characteristics are also adjoined by the musculature of the right upper limb. The jiu-jitsu group contains two clusters with two elements in each. These reflect the strong similarity between the musculature of the upper and lower limbs. In bodybuilders, the lowest agglomerative coefficient is connected to the muscle mass of their lower limbs, adjoined to the musculature of the right and left upper limb. In the control group, the analyzed characteristics behave somewhat differently. The lowest agglomeration coefficients are assigned to the muscle mass of the left lower limb and left upper limb and right lower limb. Further in the graph, these characteristics are also adjoined by the musculature of the right upper limb.

The agglomerative coefficient of the four-element cluster consisting of the variables of muscle mass of the right and left upper and lower limb and the cluster of grip strength form on a similar level for bodybuilders, judo competitors, and the control group. Somewhat weaker connection occurs in the jiu-jitsu group. The highest agglomerative coefficient is connected to the cluster of trunk musculature. Highest noted agglomerative coefficient was found in the jiu-jitsu group and the lowest one in the non-sportsmen.

## 4. Discussion

The study examined the impact of practicing various martial arts and of strength training on the asymmetry of body musculature and isometric strength of the academic judo and jiu-jitsu competitors and bodybuilders. We have also analyzed the morphofunctional structure in these groups of sportsmen and in the control group.

Especially in the contact sports, the competitor’s size is of particular importance. It is marked by four morphological factors: Height, body mass, body build, and body composition [33]. Lowest body mass was found in the subjects from the control group. Their body shape, assessed through BMI, was also the most slender. The analysis confirmed that in the men who do not practice sports, the contribution of fat in the overall body mass is the highest [34]. The percentage volume of the muscles was lowest in the group of amateur bodybuilders, which could be caused by the lower strain during physical exercise, their relatively short sports experience, and the divergent goals of their training. In martial arts athletes, well-developed muscle mass is the foundation to their strength and force; in bodybuilding, however, hypertrophy of the skeletal muscles and greatest possible reduction of subcutaneous fat tissue allow for better display of the muscles and facilitate modelling of the body’s shape [35,36,37]. It should be noted that the differences in the size of musculature of the right and left side of the body were low, thus confirming the well-known regularity arising from bilateral asymmetry typical in humans [38]. Only the judo competitors surpassed the other groups in the size of musculature of the right and left side of the body and of the right and left limb, which can be justified by their significantly longer training experience and greater body massiveness [39,40]. Moreover, the study found that in judo, the uneven physical strain for the right and left sides of the body causes both directional asymmetry (DA) and absolute asymmetry (TA), which shows in the visible prevalence of the size of muscle mass of the right upper limb, left half of the trunk, and the left lower limb [41]. This is the result of the fact that judo competitors perform grapples with their right hand, use their left leg for balance, and their right leg as the “attacking” leg. As a result, their left leg performs more work than their right leg, which causes discrepancies in the size of musculature between the right side and the left side.

Unlike judo, jiu-jitsu involves a far more diverse range of techniques that do engage the lower limbs. This leads to the decrease in bilateral asymmetry in terms of musculature [42]. Moreover, it involves far lesser two-sided differentiation in the musculature of the lower limbs in comparison to the upper limbs. The development of muscle mass of body segments in bodybuilders achieves the most symmetry in comparison to the other groups, which is a result of the discipline’s objectives that emphasize the criteria such as, e.g., the separation of the muscles and the symmetry and proportions of particular parts of the body [43].

Among the bodybuilders and the control group, significant side-to-side differences in the grip strength were noted, which indicate the functional dominance of the right limb. Among the judo and jiu-jitsu athletes, this difference was small and statistically insignificant, which may be ascribed to the way in which the competitors engage in combat which emphasizes upper-body dynamic and static strength endurance [44]. These two traits are necessary to perform particular technique-related actions (e.g., grip domination and groundwork techniques) that allow to control the opponents during attack and defense [45]. A similar decrease in the asymmetry of grip strength was also found by other researchers [39,46,47].

The analysis of the structure of the variables used in the experiment via agglomeration method highlighted the discrepancies in the development of muscle mass in body segments as a result of varying combat techniques and training goals.

In all the analyzed groups, muscle mass in the right and left side of the trunk constituted a separate cluster and shows lowest connection to the musculature traits of the limbs, which expresses the same distinctiveness of this segment. However, many electromyographic tests have shown that the erector spinae muscles are important core muscles that control the movement patterns during walking and other various rhythmical motor tasks [48]; research has also shown that those muscles play a key role in sports [49]. This has also been found to be true in our research results. The greatest asymmetry in musculature of the trunk was found in the judo competitors and the lowest asymmetry in that regard was found in the jiu-jitsu competitors, who use more varied fight techniques.

In judo competitors, a strong relationship was visible between the musculature of the right and left lower limb, which are used to maintain balanced posture, and the left upper limb, which is used to grapple the opponent. The muscle mass of the right upper limb, which is actively used in fights, is isolated from the others. Similar morphological differences exist between the pairs of limbs in karate and fencing competitors [41,50]. These are the result of asymmetrical actions taken in both disciplines and stem from the adaptation of the tissues to the increased physical strain. Moreover, the research has shown that the asymmetries were visibly marked in the upper limbs in contrast to the lower limbs [11,41]. The morphological structure in the control group confirms what was found by other researchers as well: Natural asymmetry of the right side is greater in the right-handed individuals whereas the left side of the body is less varied in that regard [51].

Overall, physical training focused on a particular discipline influences the bilateral differences in muscle mass in martial arts athletes and bodybuilders. It should be stressed that our research results represent academic sportsmen but not athletes presenting a professional level, with a longer training period and more strenuous physical effort. A significant degree of asymmetry can be found in judo competitors, whereas bodybuilders and jiu-jitsu competitors tend to be more symmetrical in terms of their musculature. In humans, a certain degree of asymmetry in the body is normal; and yet, as shown by other research, maladaptive processes that cause significant bilateral discrepancies in morphology and muscle strength of the sportsmen may lead to worse results and higher risk of incurring injuries. It was discovered that in populations of both sportsmen and non-sportsmen, the individuals with interlimb asymmetry greater than 15% are more likely to incur injuries than the individuals who are below this asymmetry percentage [52,53,54].

## 5. Conclusions

The asymmetry of body muscles and isometric strength occurs among the examined athletes. The bodybuilders tend to be more symmetrical in terms of their musculature. Jiu-jitsu contestants are less asymmetrical compared to judokas, as this discipline uses more varied techniques of lower limb fighting. So as to avoid the risk of physical trauma and negative impact on health, it is important to consistently supervise and correct the body structure in sportsmen, which also includes the symmetrical participation of the active muscle mass in particular segments. The symmetrization process should be individualized since each particular sportsman has their own side-to-side body morphology.

## Figures and Tables

**Table 1:** Statistical characteristics and inter-group differences of the morphological traits in judo competitors (J), jiu-jitsu (JJ) competitors, bodybuilders (BB), and control (C) group (SD—standard deviation).

Group	J	JJ	BB	C	*p*
Variable	Mean ± SD	Mean ± SD	Mean ± SD	Mean ± SD	
Body height [m]	1.79 ± 0.75	1.78 ± 0.54	1.81 ± 0.61	1.79 ± 0.59	0.175
Body mass [kg]	83.5 ± 14.84 *^a^*	79.1 ± 12.51	80.8 ± 11.48 *^a^*	73.4 ± 8.20	0.001
BMI	26.0 ± 3.49 *^a^*	24.9 ± 2.94 *^a^*	24.8 ± 2.63 *^a^*	22.9 ± 1.81	0.000
% Fat mass	15.3 ± 5.89 *^a,b^*	16.4 ± 4.58	15.7 ± 4.11 *^a^*	18.8 ± 4.26	0.008
% Muscle mass	62.9 ± 5.37 *^c^*	62.5 ± 4.12 *^c^*	60.6 ± 3.40	62.3 ± 4.10 *^c^*	0.000
Muscle body R [kg]	25.8 ± 4.52 *^a^*	24.9 ± 3.90	25.0 ± 3.07	22.8 ± 2.52	0.013
Muscle body L [kg]	26.4 ± 3.80 *^a^*	24.3 ± 3.20	24.4 ± 3.23	22.8 ± 3.13	0.001
Muscle arm R [kg]	4.5 ± 1.38 *^a^*	3.8 ± 1.41	3.9 ± 1.25	3.3 ± 1.12	0.008
Muscle arm L [kg]	4.2 ± 1.33 *^a^*	3.7 ± 1.25	3.8 ± 0.90	3.1 ± 0.83	0.002
Muscle leg R [kg]	8.9 ± 2.83 *^a^*	7.9 ± 2.36	7.8 ± 1.90	6.7 ± 1.60	0.003
Muscle leg L [kg]	9.0 ± 2.19 *^a^*	7.9 ± 2.40	7.8 ± 2.10	6.8 ± 1.63	0.001
Muscle trunk R [kg]	12.4 ± 2.41	13.2 ± 2.17	13.2 ± 1.61	12.8 ± 1.33	0.266
Muscle trunk L [kg]	13.2 ± 2.30	12.7 ± 1.68	12.9 ± 1.13	12.9 ± 1.17	0.735
% Muscle body R	49.3 ± 3.12	50.6 ± 1.75	50.6 ± 1.69	50.0 ± 1.99	0.063
% Muscle body L	50.7 ± 3.12	49.4 ± 1.75	49.4 ± 1.69	49.9 ± 1.99	0.063
% Muscle arm R	8.4 ± 1.65 *^a^*	7.5 ± 1.77	7.8 ± 1.89	7.1 ± 1.57	0.030
% Muscle arm L	7.9 ± 1.74 *^a^*	7.4 ± 1.61	7.5 ± 1.08	6.7 ± 1.15	0.007
% Muscle leg R	16.7 ± 3.53 *^a^*	15.9 ± 2.95	15.7 ± 2.20	14.6 ± 2.09	0.026
% Muscle leg L	17.2 ± 2.64 *^a^*	15.8 ± 3.11	15.6 ± 2.66	14.8 ± 2.12	0.009
% Muscle trunk R	24.1 ± 5.23 *^a^*	27.1 ± 4.66	27.1 ± 4.18	28.3 ± 4.19	0.004
% Muscle trunk L	25.6 ± 4.97 *^a^*	26.2 ± 4.41	26.3 ± 3.05	28.4 ± 2.32	0.027
Grip strength R [kg]	51.5 ± 8.81	47.4 ± 9.55	51.5 ± 9.79	50.1 ± 6.72	0.214
Grip strength L [kg]	50.4 ± 8.66	46.6 ± 8.03	46.8 ± 9.48	47.3 ± 7.63	0.274

*^a^* Significantly different from control group (*p* < 0.05). *^b^* Significantly different from jiu-jitsu group (*p* < 0.05). *^c^* Significantly different from bodybuilding group (*p* < 0.05).

**Table 2:** Directional asymmetry and absolute asymmetry in competitors and in control group (SD—standard deviation).

Group	J	JJ	BB	C	
Variable	Mean ± SD	Mean ± SD	Mean ± SD	Mean ± SD	*p*
**Directional Asymmetry**					
Muscle mass body	−0.74 ± 3.12	0.61 ± 1.75	0.59 ± 1.69	0.03 ± 1.99	0.192
Muscle mass arm	1.49 ± 5.81	0.24 ± 2.40	0.46 ± 3.14	1.39 ± 3.27	0.243
Muscle mass leg	−0.94 ± 4.81	0.34 ± 2.96	0.26 ± 2.10	−0.46 ± 2.48	0.241
Muscle mass trunk	−1.55 ± 5.85 *^a^*	0.89 ± 2.34	0.67 ± 3.01	−0.26 ± 2.95	0.048
Grip strength	0.50 ± 2.98 *^a^*	0.33 ± 1.96 *^a^*	2.44 ± 2.51	1.52 ± 1.95	0.001
**Absolute Asymmetry**					
Muscle mass body	2.12 ± 2.37	1.49 ± 1.07	1.20 ± 1.31	1.64 ± 1.09	0.313
Muscle mass arm	3.36 ± 4.94	1.67 ± 1.72	1.88 ± 2.54	2.68 ± 2.29	0.092
Muscle mass leg	2.60 ± 4.13 *^a^*	2.15 ± 2.02	1.22 ± 1.71	2.11 ± 1.33	0.035
Muscle mass trunk	3.95 ± 4.54	2.00 ± 1.47	2.12 ± 2.22	2.34 ± 1.75	0.437
Grip strength	2.22 ± 2.04	1.45 ± 1.33 *^a^*	2.77 ± 2.14	2.06 ± 1.34	0.037

*^a^* Significantly different from bodybuilding group (*p* < 0.05).
